# Young minds, deeper insights: a recap of the BMAS Summer School 2023, ranging from basic research to clinical implications of bone marrow adipose tissue

**DOI:** 10.1242/bio.060263

**Published:** 2024-01-30

**Authors:** Tânia Amorim, Drenka Trivanovic, Andrea Benova, Hongshuai Li, Michaela Tencerova, Biagio Palmisano

**Affiliations:** ^1^ Neuroendocrinology Unit, Division of Endocrinology and Metabolism, Department of Medicine, University of Pittsburgh School of Medicine, Pittsburgh 15206, USA; ^2^ Group for Hematology and Stem Cells, Institute for Medical Research, University of Belgrade 11000, Serbia; ^3^ Laboratory of Molecular Physiology of Bone, Institute of Physiology of the Czech Academy of Sciences, Prague 14220, Czech Republic; ^4^ Department of Orthopaedics & Rehabilitation, Carver College of Medicine, University of Iowa, Iowa City 52246, USA; ^5^ Department of Radiology, Oncology and Pathology, Sapienza University of Rome, Rome 00158, Italy

**Keywords:** Skeletal stem cells, Adipocytes, Bone marrow stromal cells, Career development, Young investigators

## Abstract

Bone marrow adiposity (BMA) is a rapidly growing yet very young research field that is receiving worldwide attention based on its intimate relationship with skeletal and metabolic diseases, as well as hematology and cancer. Moreover, increasing numbers of young scientists and students are currently and actively working on BMA within their research projects. These developments led to the foundation of the International Bone Marrow Adiposity Society (BMAS), with the goal to promote BMA knowledge worldwide, and to train new generations of researchers interested in studying this field. Among the many initiatives supported by BMAS, there is the BMAS Summer School, inaugurated in 2021 and now at its second edition. The aim of the BMAS Summer School 2023 was to educate and train students by disseminating the latest advancement on BMA. Moreover, Summer School 2023 provided suggestions on how to write grants, deal with negative results in science, and start a laboratory, along with illustrations of alternative paths to academia. The event was animated by constructive and interactive discussions between early-career researchers and more senior scientists. In this report, we highlight key moments and lessons learned from the event.

## Teaser

**Summary:** Given the growing interest in bone marrow adiposity (BMA), the International Bone Marrow Adiposity Society (BMAS) Summer School 2023 was envisioned to share advancements on BMA, and offer advice on career development to early-career investigators.

## Introduction

Bone marrow adipose tissue (BMAT) is a tissue that differs from white adipose tissue (WAT) in terms of function, structure, and location (Suchacki et al., 2020), and can account for approximately 8% of total body fat. It is composed of bone marrow adipocytes (BMAds), which play major roles in several physiological processes, such as whole-body energy metabolism, bone homeostasis, and hematopoiesis. Studies in both humans and animal models have shown the existence of two types of BMAT based on their morphology, lipid composition and function: constitutive BMAT (cBMAT) and regulated BMAT (rBMAT) (Scheller et al., 2015). cBMAT is mostly found in the extremities of the appendicular skeleton and is composed by unsaturated lipids, whereas rBMAT is found in regions with active hematopoiesis, i.e. in the axial skeleton, and is composed by saturated lipids that respond to external stimuli (Scheller et al., 2015). Recent studies have shown that BMAT changes its dynamics in response to nutritional fluxes, aging, and exercise. Furthermore, BMAT is altered in several metabolic conditions such as diabetes, osteoporosis and cancer (Veldhuis-Vlug and Rosen, 2018), and, therefore, understanding the role and function of this unique tissue could be critical for the treatment and prevention of these diseases. The International Bone Marrow Adiposity Society (BMAS) was founded with the goal of advancing our understanding of the role of BMAT in relation to bone health, hematopoiesis, cancer, systemic metabolism, and other fields (Corsi et al., 2019). BMAS is unique in encouraging collaboration between biomedical scientists and clinicians from different backgrounds by organizing meetings and specialized working groups (Tratwal et al., 2020; Bravenboer et al., 2020). Furthermore, BMAS is committed to supporting early-career researchers in the field, and for this reason the society created a program for young investigators – the BMAS Summer School. The first summer school was held in 2021, and offered a series of lectures, workshops and career development sessions, targeted to the needs of young investigators (Labella et al., 2022). Inspired by the success of the first summer school, BMAS developed the second summer school, held online during the 2023 summer. The BMAS Summer School 2023 was set up to foster the education of basic researchers and clinician scientists. Specifically, the BMAS Summer School 2023 aimed at providing: 1) general knowledge on BMAT; 2) an overview on the most state-of-the-art contemporary methodologies in BMAT related research; 3) essential training in early-career development; and 4) networking opportunities with experts, established investigators, and young scientists, aimed at fostering collaborations in the BMAT field.

To fulfill its aims, the BMAS Summer School 2023 delivered topics relevant to BMAT research (basic, translational, and clinically related topics), alongside interactive workshops that incorporated personal and professional development sessions. Moreover, the program integrated poster presentations from the submitted abstracts (Palmisano and Tencerova, 2024), and several networking sessions to provide opportunities for attendees to network with senior experts, as well as with other young researchers and clinicians.

## Highlights from the BMAS Summer School 2023

### Basic implications of BMAT

The meeting featured distinguished keynote speakers who delved into the forefront of BMAT-related research, providing comprehensive background education along with cutting-edge advancement in this intriguing field.

Prof. Christian Wolfrum (ETH Zürich, Switzerland) opened the meeting with a remarkable lecture on the “Physiology of white, brown, and beige adipocytes”. His presentation elucidated adipocyte plasticity under cold stimulation, and the heterogeneity of adipose tissue depots and its implications for metabolic functions. These insights laid a solid foundation for the exploration of the unique metabolic role of BMAT as well. Dr. Alexander Rauch (University of Southern Denmark, Denmark) explained the transcriptional control of marrow adipogenesis in his talk “Delineation of transcriptional control of marrow adipogenesis”. Dr. Rauch underscored the plasticity of bone marrow stromal cells (BMSCs) during osteogenic and adipogenic differentiation *in vitro* and showed that BMAT may affect the bone phenotype via different mechanisms under caloric restriction or high-fat diet, as two conditions that can cause BMAT expansion *in vivo*. The talk from Prof. Pamela Gehron Robey [National Institute of Health (NIH), United States], “Skeletal stem cells niches and BMAT” provided a great overview about skeletal stem cell (SSC) niches and site-specific SSCs with different profiles of cell surface markers for their characterization. She introduced the concept of tissue-specific SSC, highlighting the reasons why the term “mesenchymal stem cell” should be avoided and abandoned (Bravenboer et al., 2020). Further, Prof. Robey presented her preliminary single-cell RNAseq data from human SSCs and their molecular signature. At the end of her talk, she addressed SSC fate in relation to different pathophysiological conditions (mutations or inflammation). Specifically, she explored the role of these stem cells in various diseases and disorders (Palmisano et al., 2022), and discussed factors influencing their fate, including telomere biology disorders (TBD), *Toxoplasma gondii*-induced inflammation (Chou et al., 2012), and erythropoietin signaling manipulation (Suresh et al., 2019).

**Fig. 1.**
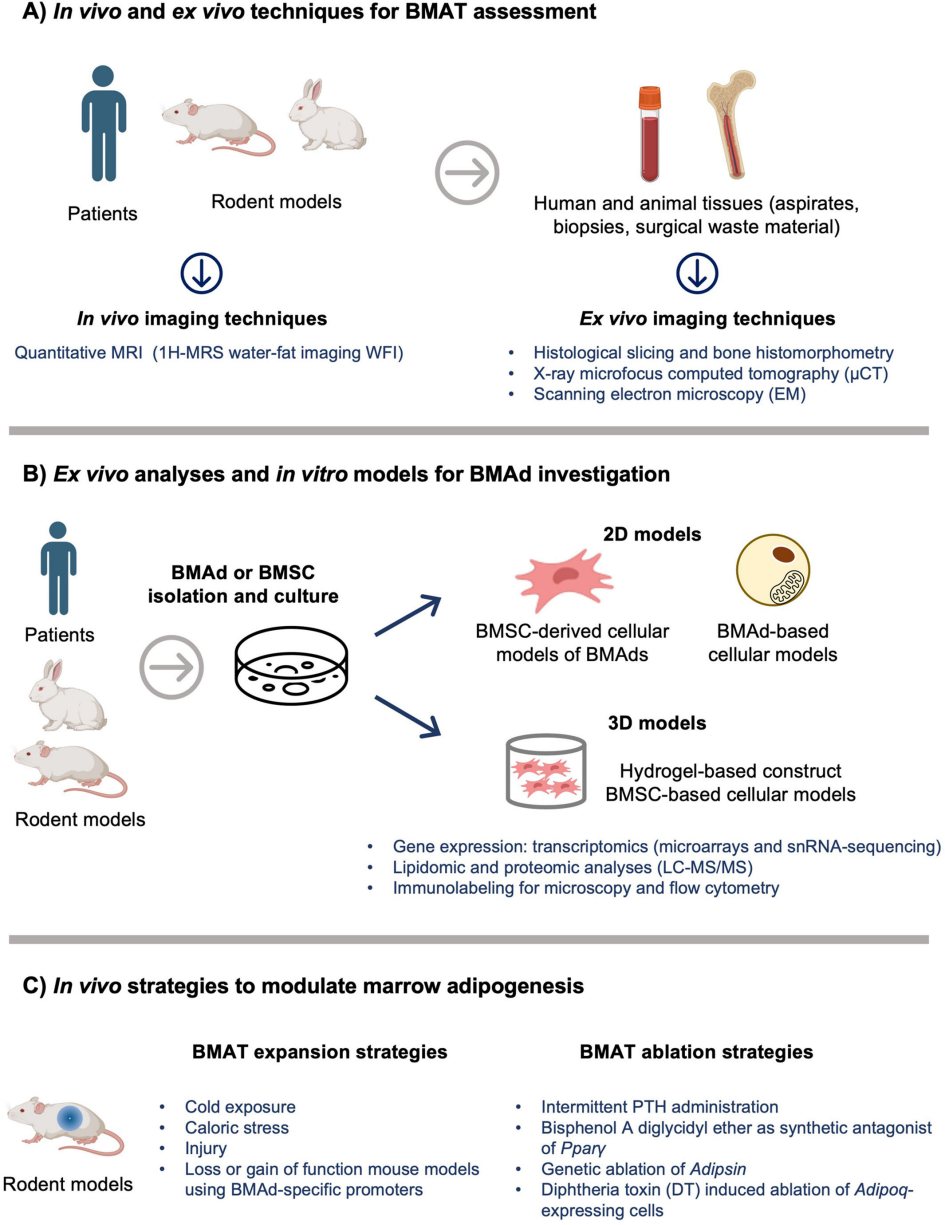
**Illustration of currently used techniques and methods to study BMAT in human and animals.** (A) Assessment of BMAT *in vivo* with non-invasive quantitative MRI for analyses of BMAT can be used for obtaining bone marrow fat fraction (BMFF) maps, the proton-density fat fraction (PDFF, the ratio of unconfounded fat signal and the unconfounded fat and water), signal fat fraction (SFF) and degree of lipid unsaturation level. Tissues from patients and animal models can be obtained and *ex vivo* analyses such as histomorphometry and µCT can be performed to assess BMAd parameters. (B) *Ex vivo* analyses and *in vitro* modelling of BMAds. Purification of BMAds is a critical step in downstream BMAd analyses. When BMAd purification is not possible, BMSCs can be isolated and induced toward adipogenic differentiation. Single-nuclei RNA sequencing (snRNA-seq) and quantitative liquid chromatography-tandem mass spectrometry (LC-MS/MS) can be applied for analyses of transcriptome, proteome, and lipid profile of BMAds. (C) Approaches to follow and modify marrow adipogenesis *in vivo*. Cold exposure, caloric stress (high-fat diet, caloric restriction, fasting), injuries can be applied to induce BMAT expansion, while PTH injections or genetic manipulations can be used to induce BMAT ablation. Figure created with BioRender.com.

Prof. Ormond A. MacDougald (University of Michigan Medical School, USA) provided the most recent findings into animal models for studying BMAT. He highlighted the pros and cons of various models, including rabbits, rats, and mice. Excitingly, he introduced a newly developed BMAd-specific *Cre* mouse model (Palmisano et al., 2022) (Fig. 1C), which provides a specific driver to modulate gene expression in BMAds without affecting other fat depots and bone. Prof. Maya Styner (University Of North Carolina, USA) in her talk “Exercise, diet and BMAT” examined a poorly understood relationship between BMAT and bone health using exercise mouse models. Despite reduction of BMAT, her studies showed that exercise can be detrimental for bone during caloric restriction in mice (McGrath et al., 2020).

Together, these lectures covered a comprehensive exploration of BMAT-related research, highlighting the diversity and depth of investigations in this dynamic field.

### Clinical relevance of BMAT

The meeting also featured presentations about the clinical relevance of BMAT.

Dr. Pouneh Fazeli (University of Pittsburgh School of Medicine, USA) with the talk “Hormonal regulation of BMAT”, provided a nice overview on BMAT regulation in different physiological and pathophysiological conditions such as starvation and anorexia nervosa. Dr. Fazeli presented the data of her recent work on the modulation of BMAT in different clinical settings, in which BMAT seems to have a different function in states of nutrient insufficiency compared to nutrient sufficiency (Fazeli et al., 2021).

Prof. Miriam A. Bredella (New York University, USA), with the talk “BMAT imaging”, gave a comprehensive introduction on BMAT imaging in humans using 1H-MRS or dual-energy computed tomography (CT), introducing the possibility of measuring saturation index in bone marrow fat fraction in relation to fracture risk. At the end of her talk, Prof. Bredella presented data on the clinical studies with weight loss surgery in adolescents and its impact on bone and BMAT, along with an overview of several clinical cases with serous atrophy of bone marrow occurring most commonly in patients with anorexia nervosa and cachexia (Boutin et al., 2015).

The lecture by Prof. Susanta K. Hui, (City of Hope National Medical Center, USA) “BMAT and malignancies” covered the topic of BMA in the context of cancer. Prof. Hui presented his work on imaging-based assessment of changes in bone marrow composition due to radiation and chemotherapy in the treatment of different types of hematological malignancies. Importantly, he pointed out advantages and drawbacks of different imaging systems for BMAT assessment in hematological malignancies (Arentsen et al., 2017). He concluded his talk sharing some of his most recent data from clinical trials where measurement of BMAT helped in the diagnosis of the progression of malignant diseases.

### Methodologies to study BMAT

Studying BMAT has many complications as this tissue is hard to access in the skeleton of animals or humans. Several lectures during the summer school discussed different approaches to study BMAT. Prof. Miriam A. Bredella provided a comprehensive introduction about the characteristics of BMAT applying various imaging techniques, including magnetic resonance imaging (MRI), CT, X-ray topography (XRT), and dual energy CT (Fig. 1A). She shared valuable insights into BMAT profiles in diverse pathophysiological conditions. Dr. Camille Attané (University of Toulouse, France), shared important insights on isolating adipocytes from bone marrow, providing protocols, recommendations, and key steps for human BMAd isolation (Lucas et al., 2021). She also discussed methods to assess BMAd post-isolation purity and viability post-isolation (Fig. 1B). On the other hand, Prof. Greet Kerckhofs (KU Leuven, Belgium) showed novel approaches to visualization of BMAT. In her talk “New tools in bone microstructure analysis - contrast enhanced computer tomography”, she highlighted *ex vivo* microCT and contrast-enhanced microCT imaging for X-ray-based 3D histology of biological tissues (Fig. 1A). Her talk focused on 3D visualization and quantification of bone microstructure, vasculature, and BMAT, utilizing contrast stains such as osmium tetroxide (OSO_4_), Hafnium-based Wells-Dawson polyoxometalate (Hf-WD POM) (Kerckhofs et al., 2018), Hexabrix/Telebrix (Benova et al., 2022) and Lugol's iodine.

## Career development sessions

Many of the attendees, such as PhD students, form career development ideas based on their academic experience. We organized sessions in the form of interactive talks, which enabled direct communication between the early-career researchers on the one hand and industry experts, recognized scientists and Principal Investigators (PIs) on the other.

During the BMAS Summer School 2023 we arranged two career development sessions focused on “Academia” and “Industry” and a career development workshop. For the “Academia” talks, we invited Dr. Alexander Rauch (PI at the University of Southern Denmark), then Dr. Konstantin Horas (orthopedic surgeon at the University of Wuerzburg, Germany) and Dr. Pouneh Fazeli (PI at the University of Pittsburgh School of Medicine, USA). Participants discussed positions and mentor selection, professional and personal life balance, and resourcing. The career path “Industry” was led by Dr. Ethan Sarnoski (Cambrian Biopharma Inc, USA). This session provided a very inspiring discussion between the lecturer and attendees in which most of the raised points were related to financial motivation, the difference in an industry-way of thinking and acting, but also possible moves back to academia from industry. The discussion pointed out all the potential advantages and limitations of the work in industry. We concluded that this session was informative for the majority of participants as most of them possessed experience from academia only. This turned out to be of great importance for career development and decision-making situations by early investigators.

During the career development workshop *“*Establish your own laboratory*”*, Dr. Michaela Tencerová (Czech Academy of Sciences*,* Czech Republic) and Dr. Thomas Ambrosi (University of California Davis, USA) gave talks discussing their outstanding and exemplary paths. Attendees had an opportunity to obtain relevant input related to career advancement and the importance of international and multidisciplinary research experience, gradual career development, mentoring, and finding objective elements (including funding) for the research team or group establishment.

In the BMAS Summer School 2023 we also organized several networking activity sessions. Prof. Miriam A. Bredella shared her experience in the “How to deal with negative results*”* topic*.* She and attendees discussed the importance of keeping objective thinking in research, pointing out the necessity to publish and interpret results even if they might not be in accordance with the hypothesis or expectation. Also, Prof. Bredella provided several very illustrative and positive examples of ‘negative’ results that led to great medical solutions. This session appeared to be very informative for early-career researchers who deal with challenging experimental work, scientific writing practice and achieving project aims.

In another networking activity, Dr. Michaela Tencerová, Dr. Thomas Ambrosi, and Prof. Ormond MacDougald gave talks on how to reach independence in science. The trio discussed the importance of persistence and objective thinking with the attendees. We concluded that both the career development and networking sessions were very valuable for attendees as well as for the organizers, both composed of PhD students, postdocs and young PIs.

## Sustainability in scientific meetings

Without a doubt, in-person meetings have many advantages compared to virtual conferences, foremost among them is the opportunity to interact with people starting from the moment you sit down for breakfast, along with easy collaboration through face-to-face communication and discussion. The amount of waste produced during in-person conferences, the carbon footprint associated with traveling, and the enormous cost needed to attend, makes events un affordable for some, especially for people from low- and middle-income countries. This is pushing us to reflect and think of new ways to organize conferences, allowing for the same social interaction and guaranteeing the same scientific dissemination in a more sustainable and environmentally friendly way.

**Fig. 2.**
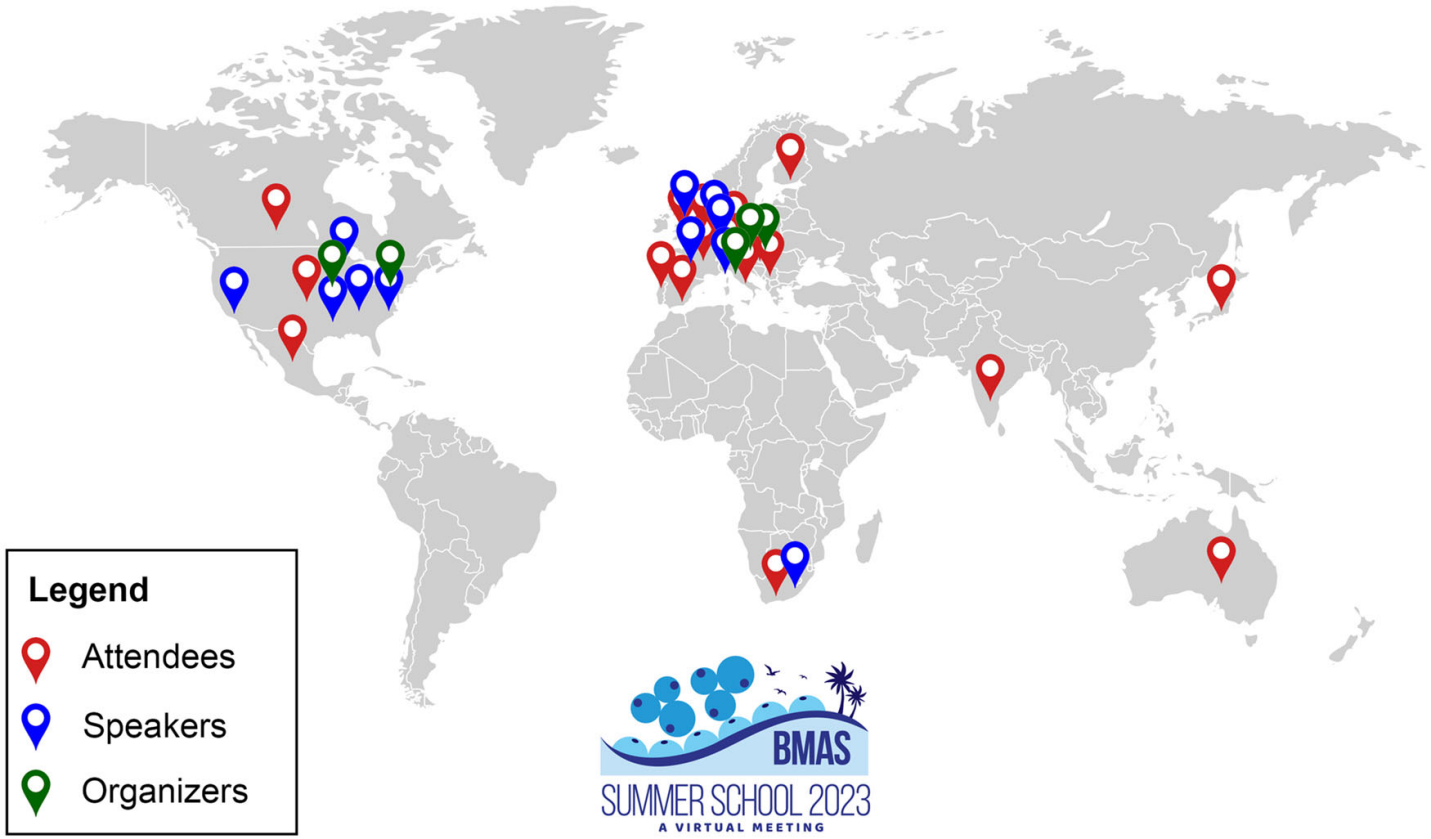
**The geography of the BMAS Summer School 2023.** Figure created with BioRender.com.

As with the first edition in 2021 (Labella et al., 2022), BMAS Summer School 2023 was a fully virtual event, which allowed for the participation of attendees from 21 different countries (Fig. 2). Invited speakers were chosen in order to ensure gender and geographic diversity and selected based on their expertise in the specific subject without considering career stage (Fig. 2). The scientific program, flyers, and other documents were paperless. All this was done in view of maximizing social sustainability and minimizing environmental impact.

## Dissemination of BMAT knowledge to LMICs researchers

The dissemination of scientific knowledge to researchers in low- and middle-income countries (LMICs) is crucial to promote global equity in scientific advancements, as it ensures that researchers in LMICs have access to the latest findings and can actively contribute to global scientific progress. In collaboration with the International Federation of Musculoskeletal Societies (IFMRS) and BMAS, we offered five awards to LMIC researchers that allowed people from Mexico, India, South Africa and Serbia to join the event with waived registration fees, breaking down a major barrier for LMIC researchers who are usually cut off from the big conferences due to the high costs associated with traveling and attending those events.
